# Explainable Machine Learning Models for Predicting FEV_1_ in Non-Smoking Taiwanese Men Aged 45–55 Years

**DOI:** 10.3390/diagnostics15243152

**Published:** 2025-12-11

**Authors:** Chih-Yueh Chang, Dee Pei, Yen-Liang Kuo, Li-Na Lee, Chung-Ze Wu, Ta-Wei Chu, Hsiang-Shi Shen, Chun-Yen Huang, Yao-Jen Liang

**Affiliations:** 1 Division of Chest Medicine, Department of Internal Medicine, Fu Jen Catholic University Hospital, Fu Jen Catholic University, New Taipei City 243089, Taiwan; cychang627@gmail.com (C.-Y.C.); pforcekuo@gmail.com (Y.-L.K.); linalee@ntu.edu.tw (L.-N.L.); 2 School of Medicine, College of Medicine, Fu Jen Catholic University, New Taipei City 242062, Taiwan; tony751028@gmail.com; 3 Graduate Institute of Applied Science and Engineering, Fu-Jen Catholic University, New Taipei City 242062, Taiwan; meureka@gmail.com; 4 Department of Internal Medicine, Fu Jen Catholic University Hospital, Fu Jen Catholic University, New Taipei City 243089, Taiwan; peidee@gmail.com; 5 Division of Endocrinology and Metabolism, Department of Internal Medicine, School of Medicine, College of Medicine, Taipei Medical University, Taipei City 110301, Taiwan; chungze@gmail.com; 6 Division of Endocrinology and Metabolism, Department of Internal Medicine, Shuang Ho Hospital, Taipei Medical University, New Taipei City 235603, Taiwan; 7 Department of Obstetrics and Gynecology, Tri-Service General Hospital, National Defense Medical Center, Taipei City 114202, Taiwan; taweichu@gmail.com; 8 MJ Health Research Foundation, Taipei City 114066, Taiwan; 9 General Education Center, Lunghwa University of Science and Technology, Taoyuan 333326, Taiwan; 10 Department and Institute of Life Science, Graduate Institute of Applied Science and Engineering, Fu-Jen Catholic University, New Taipei City 24205, Taiwan

**Keywords:** FEV_1_, lung function, machine learning, SHAP, lactate dehydrogenase, health examination cohort

## Abstract

**Background**: Traditional regression explains only part of the variation in forced expiratory volume in one second (FEV_1_). Machine learning (ML) methods may capture nonlinear patterns beyond linear assumptions. **Methods**: We analyzed 23,943 non-smoking Taiwanese men aged 45–55 years from the MJ Health Screening Cohort. Random Forest (RF), Stochastic Gradient Boosting (SGB), and XGBoost were compared with multiple linear regression (MLR) using repeated train–test splits. Model performance was evaluated with RMSE, RAE, RRSE, and SMAPE. Shapley additive explanations (SHAP) were used to interpret variable effects. **Results**: ML models achieved slightly lower prediction errors than MLR. The most influential predictors across models were lactate dehydrogenase (LDH), body weight (BW), education level, leukocyte count, total bilirubin, and sport area. SHAP indicated negative effects of LDH and leukocyte count and positive associations for BW, bilirubin, education, and physical activity. **Conclusions**: ML approaches provided modest accuracy gains and clearer interpretability compared with MLR. Biochemical and lifestyle factors—including LDH, BW, education, inflammation markers, and physical activity—contribute meaningfully to FEV_1_ among healthy middle-aged men.

## 1. Introduction

Asthma, a prevalent chronic respiratory disease, affects 1–29% of adults worldwide [1,2]. In 2019, approximately 262 million individuals struggled with asthma, leading to 455,000 deaths in the world [3]. An estimated 24 million people lived with asthma, causing 3517 deaths in USA in 2021 according to Centers for Disease Control and Prevention.

Chronic obstructive pulmonary disease (COPD) has also become one of the top three global causes of death, with over 90% of COPD-related mortality occurring in low- and middle-income countries [4,5]. The incidence of COPD peaks around age 45 [6], corresponding to our study population, and adult-onset asthma becomes the predominant phenotype by age 50 in men [7].

Forced expiratory volume in one second (FEV_1_)—a spirometric measure of the volume of air exhaled during the first second of a forced breath—is a cornerstone parameter for lung function assessment. Conventionally, FEV_1_ prediction equations are based primarily on age and height [8,9]. However, accumulating evidence suggests that FEV_1_ is influenced by additional factors, including body composition [10], smoking [11], and environmental exposure [12].

Most prior studies have employed traditional regression models, which may fail to capture nonlinear or complex interactions. Machine learning (ML) methods can overcome these limitations by learning intricate data patterns without a priori assumptions [13]. Although ML models have been increasingly applied in pulmonary research, most prior studies have focused on mixed-age or disease-specific populations and relied on limited sets of predictors. In contrast, our study investigates a highly specific, healthy, non-smoking male cohort aged 45–55 years and incorporates a broader spectrum of demographic, biochemical, and lifestyle variables than previously examined in MJ cohort research.

Rather than predicting lung disease or reproducing reference equations, this study aims to clarify secondary, non-anthropometric determinants of FEV_1_ using interpretable ML techniques. By integrating Shapley additive explanations (SHAP) analysis with three ML models, we provide new insights into nonlinear relationships and lesser-known correlates of pulmonary function within a homogeneous midlife population.

## 2. Materials and Methods

### 2.1. Patient Selection

Some of the following content was adapted from our previous publication [14]. Data were obtained from the Taiwan MJ Cohort, an ongoing prospective health-examination study conducted by the MJ Health Screening Centers in Taiwan [15]. Each health examination includes biological and clinical indicators, such as anthropometric measurements, blood tests, and imaging studies. Participants complete a self-administered questionnaire detailing medical history, current health status, lifestyle, physical activity, sleep, and dietary habits [16].

At the time of examination, all participants provided general informed consent for future anonymous research use. The dataset is maintained by the MJ Interpretation Foundation. The data used in this research were authorized and received from the MJ Health Research Foundation and MJ Interpretation Foundation (Authorization Code: MJHRF2023007A). The interpretations and conclusions presented here are solely those of the authors and do not represent the views of MJ Health Research.

Because this was a secondary analysis of de-identified data, only a brief ethics review was required. Detailed data-collection procedures are described in the annual technical report [16]. The study protocol was approved by the Fu Jen Catholic University Institutional Review Board (IRB No.: FJUH112321) and conducted in accordance with the principles of the Declaration of Helsinki.

**Figure 1:**
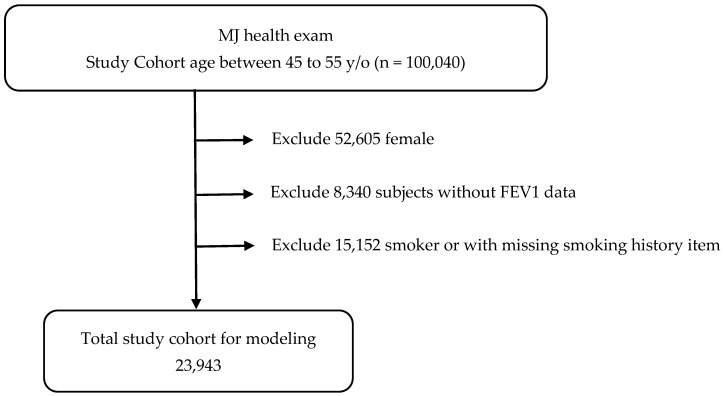
Patient selection scheme.

Initially, 100,040 subjects were identified. After applying the exclusion criteria, 23,943 non-smoking men aged 45–55 years remained available for analysis (Figure 1).

Inclusion criteria:

1. Men aged 45–55 years;
2. Never smoked;
3. Not taking medications known to affect pulmonary function;
4. Not taking medications for metabolic syndrome;
5. No major systemic or chronic diseases.

### 2.2. Measurements of Anthropometry and Biochemistry

Part of the following section has been published previously by our team [17]. On the day of examination, trained nurses recorded participants’ history of smoking, alcohol, and betel-nut use, as well as education level. Body weight (BW) was measured using a calibrated electronic scale, and chest circumference (CC) was measured horizontally at the nipple line. Systolic (SBP) and diastolic blood pressure (DBP) were measured with an automated electronic sphygmomanometer (Omron HEM-7155T, OHEM-7155T; MVN0032000 OMRON Healthcare Manufacturing Vietnam Co., Ltd. Thu Dau Mot City, Vietnam). Venous blood samples were collected after an overnight (≥10 h) fast. Plasma was separated within 1 h and stored at − 30 °C until analysis. Triglycerides (TG) were quantified using a dry multilayer analytical slide method on a Fuji Dri-Chem 3000 analyzer (Fuji Photo Film, Tokyo, Japan). High-density lipoprotein cholesterol (HDL-C) and low-density lipoprotein cholesterol (LDL-C) were measured enzymatically after dextran-sulfate precipitation.

### 2.3. Measurement of FEV_1_

Qualified paramedical technicians conducted pulmonary function testing using two identical electronic flow-type pneumotachometer spirometers (Moose PFT System; Cybermedic, Louisville, CO, USA; software version 3.8D). All tests were performed with participants seated and wearing a nose clip to prevent air leakage.

To ensure measurement accuracy, each participant completed at least three technically acceptable FEV_1_ maneuvers, with a minimum of two meeting the reproducibility criteria recommended by the American Thoracic Society (ATS) [18]. The highest FEV_1_ value among reproducible trials was used for subsequent analysis.

### 2.4. Traditional Statistical Analysis

Continuous variables are presented as means ± standard deviations (SDs). Education level, treated as an ordinal variable, was analyzed using analysis of variance (ANOVA). Pearson’s correlation was applied to examine relationships between continuous variables and FEV_1_. Multiple linear regression (MLR) was conducted as a benchmark model for comparison with ML methods. All statistical tests were two-sided, and a *p*-value < 0.05 was considered statistically significant. Analyses were performed using SPSS version 10.0 for Windows (SPSS Inc., Chicago, IL, USA).

### 2.5. Description of the Study Dataset

In this study, Table 1 summarizes the definitions and measurement methods of the 25 clinical variables included in the analysis. Because age and height are already embedded in conventional FEV_1_ prediction equations, these two variables were excluded from model development.

The variables were grouped into three major categories:

1. Demographic: BW, CC, SBP, DBP, and education level.
2. Biochemical: leukocyte count, hemoglobin, platelet count, total bilirubin, total protein, albumin, aspartate aminotransferase (AST), alanine aminotransferase (ALT), gamma-glutamyltransferase (γ-GT), LDH, creatinine, uric acid, TG, HDL-C, LDL-C, thyroid-stimulating hormone (TSH), and C-reactive protein (CRP).
3. Lifestyle: drinking habits and sport area.

Drinking habits were classified as non-drinkers or drinkers. The sport area was calculated by multiplying exercise duration, frequency, and intensity to represent overall physical activity. All parameters listed above served as independent variables, whereas FEV_1_ was the dependent variable.

### 2.6. Data Preprocessing, Model Validation and Sensitivity Analyses

Missing values were assessed across all predictors. Because the overall proportion of missingness was low (<3% for most variables, with only sport area exceeding 10%) and shown as Supplementary Materials Table S1, a complete-case analysis was applied, and the final analytic dataset included only participants with complete information.

Continuous predictors were visually inspected for extreme values using histograms and boxplots; observations exceeding ± 4 standard deviations were winsorized to reduce undue influence while preserving sample size. Right-skewed biochemical markers (e.g., LDH, γ-GT, TG, CRP) were log-transformed to improve symmetry and stabilize variance.

To minimize overfitting, all ML models were trained using 10-fold cross-validation with internal hyperparameter tuning performed within each fold to prevent data leakage. Model performance metrics were averaged across folds. Because no independent dataset of healthy Taiwanese men aged 45–55 was available, external validation could not be conducted and is acknowledged as a limitation. The study therefore focuses on internal validation to assess robustness and generalizability within this population.

To evaluate the robustness of our findings, we repeated the entire modeling pipeline under several alternative specifications, including (i) adding age and height as predictors, (ii) switching the data split from 80/20 to 70/30, (iii) replacing 10-fold CV with 5-fold CV, and (iv) removing both winsorization and log-transformation steps. Across all scenarios, RMSE differences remained within a small tolerance (ΔRMSE < 0.05 across preprocessing variations), and most of the Wilcoxon signed-rank test *p*-values for ML vs. MLR remained within the same interpretation category (*p* < 0.05) summarized as Supplementary Materials Table S2. These findings indicate that the model performance and comparative conclusions were stable regardless of reasonable changes in preprocessing or validation strategy.

### 2.7. Proposed Machine Learning Scheme

We developed predictive models to identify and rank factors associated with FEV_1_ using three distinct ML methods. Part of the methodological framework has been reported previously by our research team [14].

The first algorithm applied was the Random Forest (RF) model, an ensemble learning approach based on decision trees. RF combines bootstrap resampling and bagging [19]. It constructs multiple classification and regression trees (CART) using randomly selected subsets of the data and predictors, employing Gini impurity reduction as the splitting criterion. Predictions from individual trees are then aggregated by averaging (for regression) or voting (for classification), resulting in a robust final model [20].

The second method was Stochastic Gradient Boosting (SGB), a tree-based boosting algorithm that integrates both bagging and boosting techniques to reduce overfitting [21]. In SGB, weak learners (typically shallow decision trees) are sequentially built so that each successive tree focuses on the residual errors of the previous one. This process continues iteratively until the model converges or reaches a predefined stopping criterion. The ensemble of trees collectively contributes to the final, high-performing model.

The third model was eXtreme Gradient Boosting (XGBoost), an advanced implementation of gradient boosting that improves computational efficiency and prediction accuracy [22]. XGBoost employs a second-order Taylor expansion to approximate the objective function and integrates regularization terms to control model complexity, thereby mitigating overfitting [23].

While these ML methods effectively identify important predictors, they do not directly indicate whether each variable exerts a positive or negative influence. To address this limitation, Shapley Additive Explanations (SHAP) analysis was applied to the XGBoost model. SHAP is based on cooperative game theory and quantifies each feature’s contribution to individual predictions by comparing model outputs with and without the feature across all possible feature combinations [24,25,26]. This approach provides both a ranking of feature importance and the directionality (positive or negative) of each feature’s effect.

**Figure 2:**
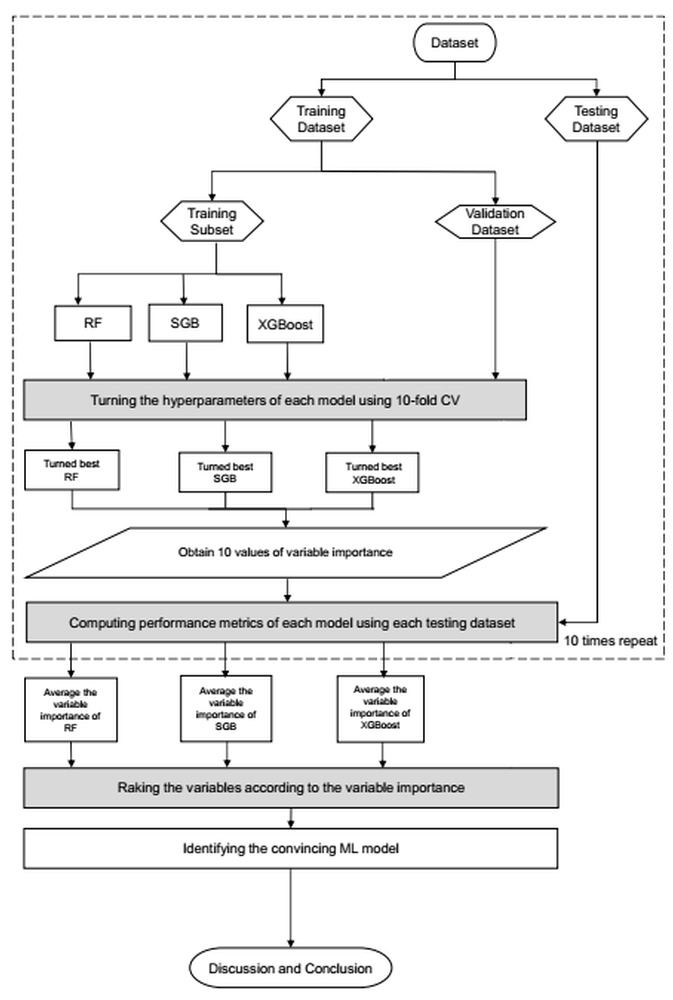
Proposed machine learning prediction scheme.

Figure 2 illustrates the overall ML workflow. The dataset was randomly divided into 80% training and 20% testing subsets. During model development, models were tuned using 10-fold cross-validation with a modest grid of hyperparameters. The model achieving the lowest root mean square error (RMSE) on the validation folds was selected as optimal for each algorithm (RF, SGB, and XGBoost). We added the full hyperparameter search space and the final selected values for RF, SGB, and XGBoost in Supplementary Materials Table S3 to improve transparency and reproducibility.

In the testing phase, model performance was evaluated using RMSE as the primary accuracy metric. Although other regression metrics such as symmetric mean absolute percentage error (SMAPE), relative absolute error (RAE), and root relative squared error (RRSE) exist, they were not applied here, as they are less commonly used in pulmonary function prediction and were not part of our prespecified analysis plan [27,28,29,30,31]. The specific metric values can be found in Table 2.

All ML algorithms generated feature importance rankings, which were compared across models to identify consistent predictors. In the final analytical phase, SHAP was used to quantify both the relative importance and directionality of each variable’s contribution to the predicted FEV_1_.

All analyses were performed using R software (version 4.0.5) with RStudio (version 1.1.453), and relevant R packages (available at www.R-project.org and RStudio). SHAP analyses were conducted in Python 3.10 using the shap, pandas, numpy, and matplotlib libraries for computation and visualization, including summary, bar, and waterfall plots.

## 3. Results

A total of 23,943 men were included in the final analysis (Figure 1). Table 1 summarizes participant characteristics. The mean age was 49.6 ± 3.2 years.

From the Pearson correlation analysis (Table 3), FEV_1_ showed significant negative correlations with leukocyte count (r = −0.161, *p* < 0.0005), SBP (r = −0.150, *p* < 0.0005), and LDH (r = −0.337, *p* < 0.0005). Positive correlations were observed with BW (r = 0.156, *p* < 0.0005), total bilirubin (r = 0.146, *p* < 0.0005), HDL-C (r = 0.136, *p* < 0.0005), and education level (r = 0.298, *p* < 0.0005).

Approximately 64% of participants held a higher education degree (college, university, or postgraduate). Significant differences in FEV_1_ were observed across educational levels, specifically between primary and junior high, junior and senior high, senior high and college, and between university and postgraduate levels (*p* < 0.0005). Most participants were non-drinkers (78.3%), and FEV_1_ differed significantly between drinkers and non-drinkers (*p* < 0.0005).

All three ML models—RF, SGB, and XGBoost—produced slightly lower prediction errors than the traditional MLR benchmark. RF (SMAPE = 0.1446, RAE = 0.1381, RRSE = 0.1381, RMSE = 0.8778), SGB (SMAPE =0.1429, RAE = 0.1370, RRSE = 0.8582), XGBoost (SMAPE =0.1426, RAE = 0.1369, RRSE = 0.8576), and MLR (SMAPE = 0.1460, RAE = 0.1460, RRSE = 0.8762) shown as Table 4.

Across repeated train–test partitions, all three ML models showed lower mean RMSE than the MLR baseline (Table 5). Paired Wilcoxon signed-rank tests further confirmed that each ML model achieved significantly lower RMSE compared with MLR (RF: *p* = 0.0039; SGB: *p* = 0.0019; XGBoost: *p* = 0.0019). Although these results demonstrate statistically consistent improvements, the absolute performance gains were modest, indicating that the clinical significance of these differences should be interpreted with caution.

**Figure 3:**
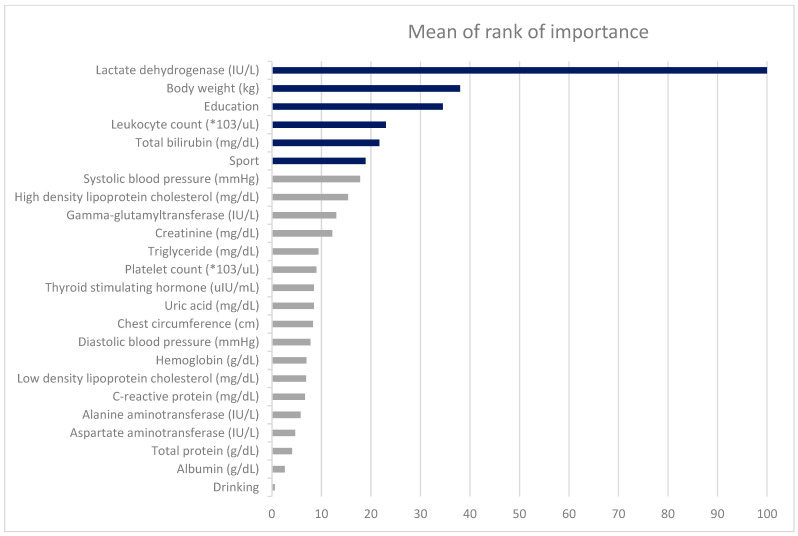
The ranks of the factors derived from four different machine learning methods.

Table 6 presents the variable importance scores across ML methods, with the averaged rankings displayed in the rightmost column. The six most influential predictors of FEV_1_ were LDH, BW, education level, leukocyte count, total bilirubin, and sport area. The relative importance rankings from each model are visualized in Figure 3.

**Figure 4:**
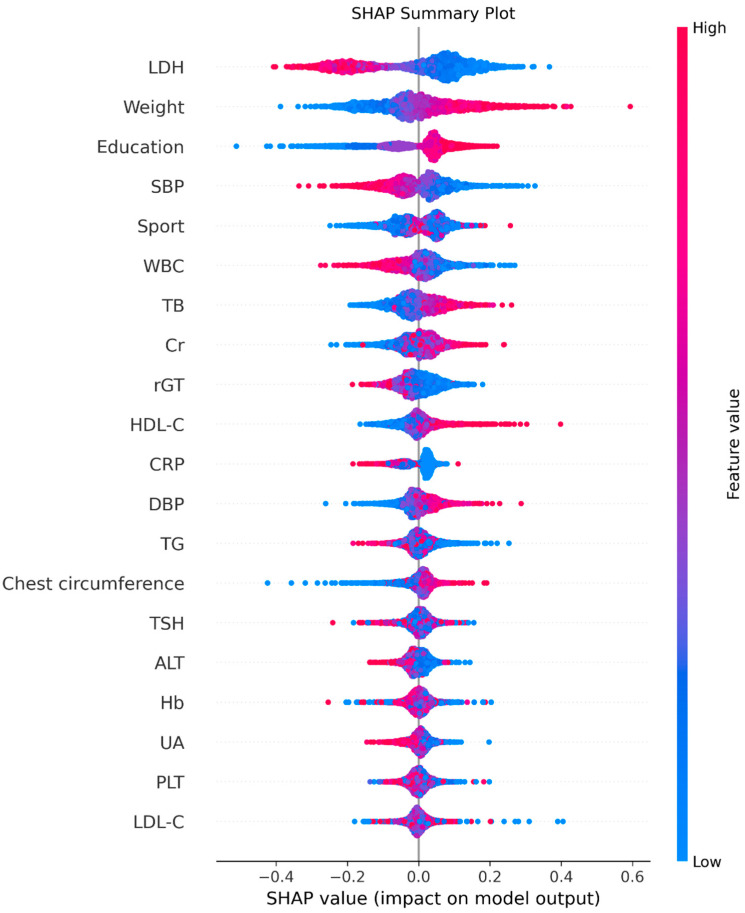
The summary plot. Red indicates higher feature values and blue indicates lower values. Points positioned to the right reflect positive contributions to FEV_1_, and those to the left reflect negative contributions.

As detailed in the Methods, SHAP analysis was applied to quantify each variable’s contribution and direction of effect on the XGBoost model. Figure 4 (SHAP summary plot) depicts the overall feature contributions across all participants: each dot represents a participant’s SHAP value for a given feature, with warmer colors (red) indicating higher feature values and cooler colors (blue) lower values. LDH emerged as the most influential variable.

**Figure 5:**
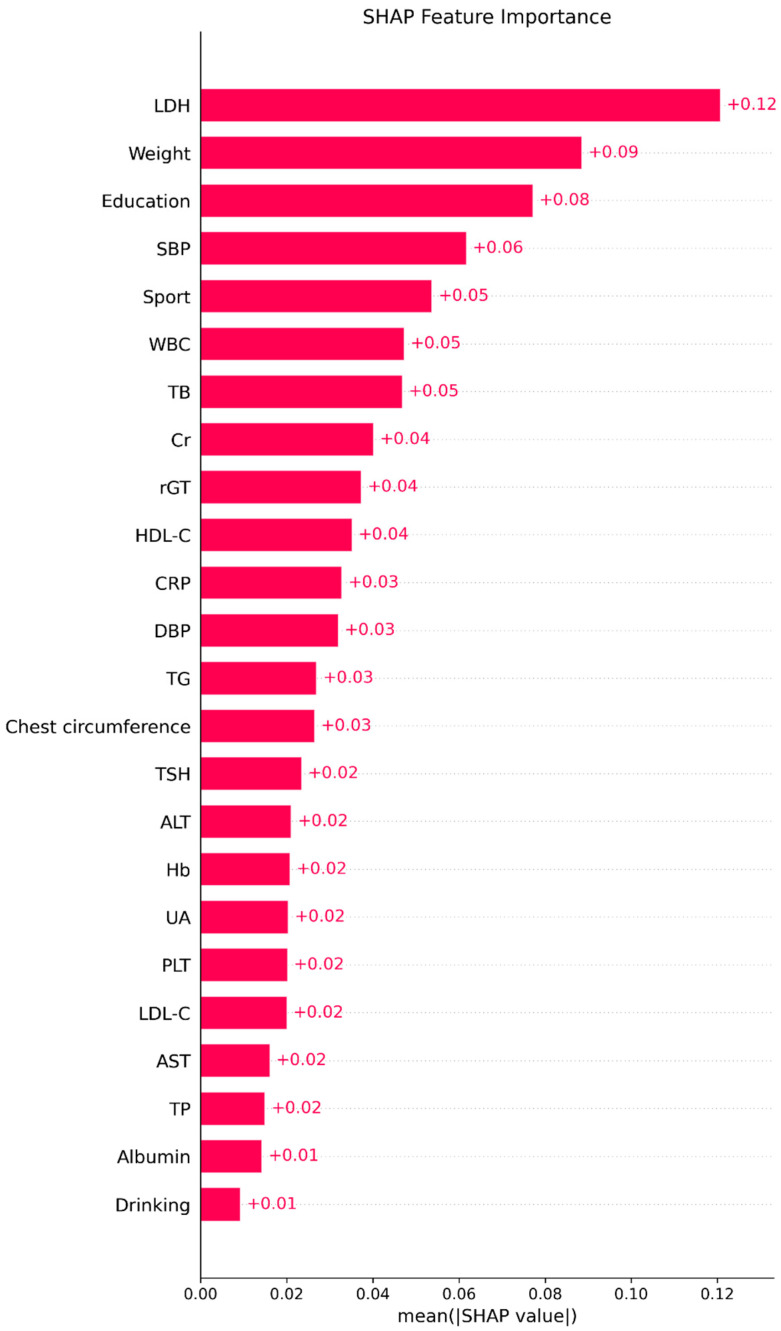
The importance plot (mean absolute SHAP values). Bars display the mean absolute SHAP value for each predictor in the final ML model, summarizing the global contribution of that variable to the model’s predictions; longer bars indicate greater overall importance.

**Figure 6:**
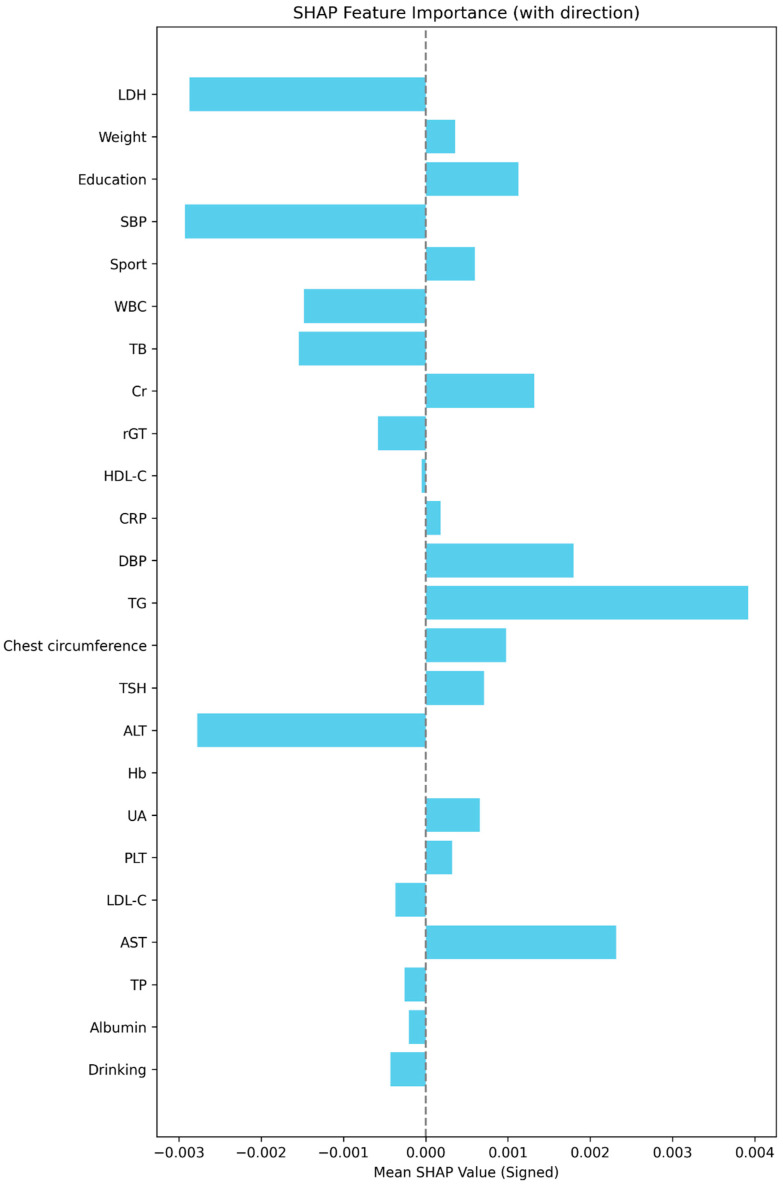
The signed SHAP value plot (mean signed SHAP values with directionality). This plot displays the mean signed SHAP value for each predictor in the final model, summarizing both the strength and average direction of each feature’s effect on the model’s predicted outcome. The dashed vertical line marks SHAP = 0. Bar length reflects effect size; color is uniform to emphasize direction rather than magnitude.

Figure 5 displays the mean absolute SHAP values, confirming the same order of global importance as Figure 4. Figure 6 presents the signed SHAP values, showing whether each variable positively or negatively influenced predicted FEV_1_. Consistent with earlier findings, LDH and leukocyte count exerted negative effects, while BW, total bilirubin, sport area, and education level had positive effects.

## 4. Discussion

This study offers a targeted examination of the factors associated with FEV_1_ in a homogeneous population of healthy, non-smoking Taiwanese men aged 45–55 years. Rather than proposing new algorithms, the novelty lies in applying established ML methods to a uniquely constrained cohort to uncover secondary biochemical and lifestyle determinants that are not emphasized in conventional lung-function equations.

Despite modest performance differences among ML models, the consistent identification of LDH, BW, education level, leukocyte count, bilirubin, and physical activity highlights patterns that traditional regression alone may not fully capture. Using SHAP for model interpretability further clarifies the direction and relative contribution of each predictor, offering a clearer understanding of subtle, nonlinear relationships influencing pulmonary function in midlife males.

Our aim was not to replicate or replace population-reference FEV_1_ equations, but rather to explore secondary determinants of lung function within a homogeneous midlife male cohort where age- and height-related variability is minimized.

LDH emerged as the strongest negative predictor of FEV_1_. Elevated LDH activity in the airways may arise from necrosis or rupture of airway or alveolar epithelial cells and activated macrophages, reflecting cellular injury and inflammation [32]. Because LDH is an intracellular enzyme present in nearly all tissues, its appearance in extracellular fluid indicates tissue damage. Consistent with our results, a cross-sectional analysis of 3453 adults reported that each 1 U/L increase in LDH was associated with a 1.11 mL decline in FEV_1_ (95% CI: −1.82 to −0.39; *p* = 0.0025) [33]. The strong negative correlation observed in our study (r = −0.337, *p* < 0.0005) reinforces the role of LDH as a sensitive and easily measurable biomarker of subclinical pulmonary injury.

BW ranked as the second most influential factor. The relationship between body composition and lung function has not been widely explored in healthy populations. A Korean study found that underweight adults exhibited lower pulmonary function, potentially due to insufficient respiratory muscle mass—particularly in the diaphragm—leading to reduced ventilatory capacity [34]. Conversely, excessive weight and obesity have been associated with adverse effects on lung function and progressive decline in FEV_1_ [35]. Our study uniquely focuses on a healthy population, revealing a modest positive correlation between BW and FEV_1_ (r = 0.156, *p* < 0.0005), suggesting that optimal muscle mass and nutritional status may contribute to better pulmonary mechanics.

Education level was the third strongest predictor of FEV_1_, reflecting the broader influence of socioeconomic status on health. In a Dutch cohort of 2679 men aged 26–66 years, those with the lowest education had FEV_1_ values 221 mL lower than those with higher education [36]. Similarly, Polak et al. [37] found that sustained high socioeconomic status throughout life was associated with greater FEV_1_ compared with persistent low SES or downward mobility. Our findings align with these results: participants with the highest education level demonstrated mean FEV_1_ values 820 mL higher than those with the lowest level. Although differences between adjacent education categories (e.g., college vs. university) were non-significant—likely due to small sample sizes—the overall trend underscores the importance of socioeconomic and behavioral factors in respiratory health. Higher educational attainment may reflect better health literacy, healthier lifestyle patterns, and improved socioeconomic conditions, which are known to influence pulmonary health through reduced exposure to smoking, better nutrition, and access to preventive care.

Leukocyte count was inversely related to FEV_1_, supporting the concept that systemic inflammation contributes to impaired lung function. Elevated leukocytes may release proteolytic enzymes and reactive oxygen species, causing tissue injury and airway remodeling [38,39]. In the U.S. NHANES III cohort (*n* = 16,312), higher leukocyte counts were independently associated with lower FEV_1_ [40]. Our findings (r = −0.161, *p* < 0.0005) corroborate this relationship, suggesting that even in non-smokers, subtle systemic inflammation may adversely influence pulmonary capacity.

Total bilirubin exhibited a positive association with FEV_1_, consistent with its recognized antioxidant and cytoprotective properties [41,42]. Bilirubin has been proposed to mitigate oxidative stress and inflammation in tissues exposed to environmental oxygen, including the lungs. In a Korean community-based cohort of 7986 adults, higher bilirubin levels were significantly associated with better FEV_1_, FVC, and FEF25–75%, particularly among never-smokers [43]. Our study extends these findings to a Taiwanese male cohort, suggesting that moderate elevations in bilirubin may confer protection against subclinical airway inflammation and decline in lung function.

Physical activity (the sport area variable)—representing exercise frequency, duration, and intensity—ranked sixth in importance. Previous studies have reported that non-smokers exhibit a dose–response relationship between exercise level and FEV_1_ [44]. For instance, men with higher physical activity had mean FEV_1_ values 154 mL greater than sedentary peers (*p* = 0.001). Other studies likewise support positive associations between fitness and lung capacity [45,46]. Higher activity levels could improve respiratory muscle strength, reduce systemic inflammation, and enhance overall cardiometabolic fitness, all of which support better FEV_1_. Although our simple correlations did not reach statistical significance, ML methods detected a latent relationship between physical activity and pulmonary performance. This suggests that nonlinear modeling may uncover subtle or interaction-dependent associations that traditional statistics overlook.

This study has several limitations. First, the cross-sectional design precludes causal inference. Future longitudinal studies are needed to verify temporal relationships and assess predictive validity. Second, the sample consisted exclusively of Taiwanese men; caution should therefore be exercised in extrapolating these findings to other ethnicities or women. Third, external validation could not be conducted, because no independent dataset of healthy Taiwanese men aged 45–55 was available. Finally, despite careful variable selection, residual confounding from unmeasured factors (e.g., environmental exposures) cannot be excluded.

## 5. Conclusions

This study contributes to the existing literature by focusing on a highly specific and clinically relevant subgroup—healthy, non-smoking Taiwanese men aged 45–55 years—which is rarely analyzed independently, despite this age range being a key turning point for the onset of adult respiratory decline.

Unlike most ML studies that evaluate mixed-age or disease-specific populations, our analysis isolates secondary biochemical and lifestyle predictors while controlling for the dominant influences of age and height. By applying explainable ML methods and SHAP analysis, the study uncovers nonlinear relationships involving LDH, leukocyte count, bilirubin, BW, and physical activity that traditional regression may understate.

These findings extend prior MJ cohort research by demonstrating that even within a narrow, homogeneous age-band, measurable biochemical signals and lifestyle behaviors meaningfully shape pulmonary function. This refined understanding highlights potential targets for early screening and health-promotion strategies in midlife men.

## Figures and Tables

**Table 1:** The demographic, biochemistry and lifestyle data of participants (*n* = 23,943).

Variable	Values (Mean ± SD)
Body weight (kg)	70.8 ± 10.2
Chest circumference (cm)	93.6 ± 6.4
Leukocyte count (*10^3^/uL)	6.0 ± 1.6
Hemoglobin (g/dL)	15.2 ± 1.1
Platelet count (*10^3^/uL)	228.9 ± 50.9
Total bilirubin (mg/dL)	1.0 ± 0.4
Total protein (g/dL)	7.5 ± 0.4
Albumin (g/dL)	4.5 ± 0.2
Aspartate aminotransferase (IU/L)	26.7 ± 16.9
Alanine aminotransferase (IU/L)	35.3 ± 29.9
Gamma-glutamyltransferase (IU/L)	35.3 ± 46.1
Lactate dehydrogenase (IU/L)	207.1 ± 79.7
Creatinine (mg/dL)	1.1 ± 0.3
Uric acid (mg/dL)	6.7 ± 1.4
Triglyceride (mg/dL)	145.4 ± 112.4
High density lipoprotein cholesterol (mg/dL)	50.7 ± 12.3
Low density lipoprotein cholesterol (mg/dL)	126.2 ± 32.8
Thyroid stimulating hormone (uIU/mL)	1.6 ± 1.6
C-reactive protein (mg/dL)	0.2 ± 0.5
Systolic blood pressure (mmHg)	124.3 ± 16.9
Diastolic blood pressure (mmHg)	79.4 ± 11.4
Sport hour/week (hour)	9.0 ± 9.1
Education	
Illiterate	72 (0.3%)
Primary school	1797 (7.7%)
Junior high school	1713 (7.3%)
Senior high school	4851 (20.8%)
College	5131 (22.0%)
University	5819 (25.0%)
Higher than master’s degree	3937 (16.9%)
Drinking	
Non-drinker	17,906 (78.3%)
Drinker	4977 (21.7%)

**Table 2:** Equation of performance metrics.

Metrics	Description	Calculation
SMAPE	Symmetric Mean Absolute Percentage Error	$SMAPE=\frac{1}{n}\sum_{i=1}^{n}\frac{\left|y_{i}-{\hat{y}}_{i}\right|}{\left(\left|y_{i}\right|+\left|{\hat{y}}_{i}\right|\right)/2}\times 100$
RAE	Relative Absolute Error	$RAE=\sqrt{\frac{\sum_{i=1}^{n}{\left(y_{i}-{\hat{y}}_{i}\right)}^{2}}{\sum_{i=1}^{n}{\left(y_{i}\right)}^{2}}}$
RRSE	Root Relative Squared Error	$RRSE=\sqrt{\frac{\sum_{i=1}^{n}{\left(y_{i}-{\hat{y}}_{i}\right)}^{2}}{\sum_{i=1}^{n}{\left(y_{i}-{\hat{y}}_{i}\right)}^{2}}}$
RMSE	Root Mean Squared Error	$RMSE=\sqrt{\frac{1}{n}\sum_{i=1}^{n}{\left(y_{i}-{\hat{y}}_{i}\right)}^{2}}$

**Table 3:** The results of Pearson correlation between baseline demographic, biochemistry, lifestyle and FEV1. BW: Body weight; CC: Chest circumference; WBC: White blood cell; Hb: Hemoglobin; PLT: Platelet count; TB: Total bilirubin; TP: Total protein; AST: Aspartate aminotransferase; ALT: Alanine aminotransferase; γ-GT: Gamma-glutamyltransferase; LDH: Lactate dehydrogenase; Cr: Creatinine; UA: Uric acid; TG: Triglyceride; HDL-C: High density lipoprotein cholesterol; LDL-C: Low density lipoprotein cholesterol; TSH: Thyroid stimulating hormone; CRP: C-reactive protein; SBP: Systolic blood pressure; DBP: Diastolic blood pressure. * *p* < 0.05, *** *p* < 0.005.

BW	CC	WBC	Hb	PLT	TB	TP	Albumin	AST	ALT	γ-GT	LDH	Cr
0.156 ***	0.091 ***	−0.161 ***	−0.005	−0.089 ***	0.146 ***	−0.096 ***	−0.073 ***	−0.052 ***	−0.048 ***	−0.059 ***	−0.337 ***	−0.015 *
**UA**	**TG**	**HDL-C**	**LDL-C**	**TSH**	**CRP**	**SBP**	**DBP**	**Sport**	**Education**	**Drinking**		
−0.095 ***	−0.086***	0.136 ***	−0.023 ***	−0.018 ***	−0.070 ***	−0.150 ***	−0.042 ***	0.035 ***	0.298 ***	−0.057 ***		

**Table 4:** The performance of linear regression and three machine learning methods.

	SMAPE	RAE	RRSE
LR	0.1460	0.1401	0.8762
RF	0.1446	0.1381	0.8662
SGB	0.1429	0.1370	0.8582
XGboost	0.1426	0.1369	0.8576

LR: linear regression; RF: random forest; SGB: stochastic gradient boosting; XGBoost: eXtreme Gradient Boosting.

**Table 5:** RMSE ± SD and 95% Confidence Intervals and Wilcoxon Signed-Rank Tests Comparing RMSE of ML Models vs. MLR.

Model	RMSE (Mean ± SD)	95% CI (Lower)	95% CI (Upper)
MLR	0.5344 ± 0.0082	0.5252	0.5437
RF	0.5321 ± 0.0077	0.5233	0.5408
SGB	0.5258 ± 0.0073	0.5176	0.5341
XGBoost	0.5252 ± 0.0074	0.5168	0.5335
**Comparison**		***p*-value**	
RF vs. MLR		0.0039	
SGB vs. MLR		0.0019	
XGBoost vs. MLR		0.0019	

**Table 6:** The rank of the importance (%) of the factors derived from linear regression and machine learning methods.

Variables	LR	RF	SGB	XGboost	MORI
Body weight (kg)	55.2	42.1	41.2	30.7	38.0
Chest circumference (cm)	0.0	16.0	5.1	3.8	8.3
Leukocyte count (*10^3^/uL)	44.6	34.3	18.5	16.3	23.0
Hemoglobin (g/dL)	10.0	17.4	1.7	1.8	7.0
Platelet count (*10^3^/uL)	8.3	22.2	2.5	2.4	9.0
Total bilirubin (mg/dL)	45.4	32.3	18.9	14.0	21.7
Total protein (g/dL)	2.4	11.8	0.4	0.0	4.1
Albumin (g/dL)	1.1	7.6	0.1	0.0	2.6
Aspartate aminotransferase (IU/L)	18.7	13.3	0.0	0.7	4.7
Alanine aminotransferase (IU/L)	18.9	16.5	0.1	0.9	5.8
Gamma-glutamyltransferase (IU/L)	21.8	22.0	9.5	7.5	13.0
Lactate dehydrogenase (IU/L)	100.0	100.0	100.0	100.0	100.0
Creatinine (mg/dL)	8.7	19.7	8.5	8.4	12.2
Uric acid (mg/dL)	19.8	19.0	3.5	2.9	8.5
Triglyceride (mg/dL)	25.6	23.0	3.2	1.9	9.4
High density lipoprotein cholesterol (mg/dL)	52.5	23.5	12.1	10.5	15.4
Low density lipoprotein cholesterol (mg/dL)	29.9	17.3	2.6	0.9	6.9
Thyroid stimulating hormone (uIU/mL)	5.2	22.4	1.0	2.2	8.5
C-reactive protein (mg/dL)	11.8	5.1	7.7	7.2	6.7
Systolic blood pressure (mmHg)	59.6	26.0	15.7	11.7	17.8
Diastolic blood pressure (mmHg)	37.5	16.9	3.9	2.6	7.8
Sport	13.8	20.5	14.4	21.7	18.9
Education	73.0	29.6	37.8	36.1	34.5
Drinking	11.0	0.0	1.2	0.6	0.6

LR: linear regression; RF: random forest; SGB: stochastic gradient boosting; XGBoost: eXtreme Gradient Boosting; MROI: mean of rank of importance. The darkness of the blue color indicates the importance of the variable. The darker the color, the more important the variable is.

## Data Availability

The data are not publicly available due to privacy restrictions.

## Supplementary Materials

The following supporting information can be downloaded at: https://www.mdpi.com/article/10.3390/diagnostics15243152/s1, Table S1: Missing Values Summary (*n* = 23,943); Table S2: Scenario Model RMSE (mean ± SD) with ΔRMSE vs. Primary; Table S3: Hyperparameter Search Space and Selected Values for Machine-Learning Models.
