# Supplementary material for: Explainable Machine Learning Models for Predicting FEV1 in Non-Smoking Taiwanese Men Aged 45–55 Years
Source: Diagnostics (Basel). 2025 Dec 11;15(24):3152. doi: 10.3390/diagnostics15243152 (PMC12731951; doi:10.3390/diagnostics15243152)
Supplement: Supplementary file 1 [file diagnostics-15-03152-s001.zip › Supplementary_Table_S1_Missing_Values.pdf]

Supplementary Table S1. Missing Values Summary (n=23,943)

Variable	Missing Count	Missing Percentage
Body weight	6	0.03%
Chest circumference	8	0.03%
Leukocyte count	4	0.02%
Hemoglobin	8	0.03%
Platelet count	4	0.02%
Total bilirubin	27	0.11%
Total protein	32	0.13%
Albumin	33	0.14%
Aspartate aminotransferase	24	0.10%
Alanine aminotransferase	23	0.10%
Gamma-glutamyltransferase	130	0.54%
Lactate dehydrogenase	984	4.11%
Creatinine	4	0.02%
Uric acid	22	0.09%
Triglyceride	5	0.02%
High density lipoprotein cholesterol	577	2.41%
Low density lipoprotein cholesterol	607	2.54%
Thyroid stimulating hormone	1845	7.71%
C-reactive protein	2131	8.90%
Education	623	2.60%
Systolic blood pressure	6	0.03%

Diastolic blood pressure	6	0.03%
Drinking	1060	4.43%
Sport_area	3878	16.20%
FEV1	0	0.00%
