# Supplementary material for: Explainable Machine Learning Models for Predicting FEV1 in Non-Smoking Taiwanese Men Aged 45–55 Years
Source: Diagnostics (Basel). 2025 Dec 11;15(24):3152. doi: 10.3390/diagnostics15243152 (PMC12731951; doi:10.3390/diagnostics15243152)
Supplement: Supplementary file 1 [file diagnostics-15-03152-s001.zip › Supplementary_Table_S2_Sensitivity Analysis.pdf]

Supplementary Table S2. Scenario Model RMSE (mean ± SD) with ΔRMSE vs Primary

Scenario	Model	RMSE (mean ± SD)	Delta_RMSE	Wilcoxon p vs. MLR
Primary pipeline (80/20, winsor + log)	MLR	0.5344 ± 0.0082	-	-
	RF	0.5321 ± 0.0077	-0.0023	0.00390625
	SGB	0.5258 ± 0.0073	-0.0086	0.001953125
	XGBoost	0.5252 ± 0.0074	-0.0092	0.001953125
70/30 split	MLR	0.4966 ± 0.0038	-0.0378	
	RF	0.4995 ± 0.0044	-0.0349	0.001953125
	SGB	0.4904 ± 0.0038	-0.044	0.001953125
	XGBoost	0.4905 ± 0.0039	-0.0439	0.001953125
No winsorization and log-transformation	MLR	0.5569 ± 0.0072	0.0225	
	RF	0.5487 ± 0.0073	0.0142	0.001953125
	SGB	0.5354 ± 0.0065	0.001	0.001953125
	XGBoost	0.5361 ± 0.0062	0.0016	0.001953125
Add in Height and Age	MLR	0.4984 ± 0.0066	-0.036	
	RF	0.5016 ± 0.0062	-0.0328	0.00390625
	SGB	0.4915 ± 0.0064	-0.043	0.001953125
	XGBoost	0.4909 ± 0.006	-0.0436	0.001953125
change CV 10 to 5	MLR =	0.5357 ± 0.0052	0.0013	
	RF =	0.5342 ± 0.0057	-0.0002	0.232421875
	SGB =	0.5274 ± 0.0053	-0.007	0.001953125

XGBoost =	0.5272 ± 0.0058	-0.0072	0.001953125
