# Supplementary material for: Explainable Machine Learning Models for Predicting FEV1 in Non-Smoking Taiwanese Men Aged 45–55 Years
Source: Diagnostics (Basel). 2025 Dec 11;15(24):3152. doi: 10.3390/diagnostics15243152 (PMC12731951; doi:10.3390/diagnostics15243152)
Supplement: Supplementary file 1 [file diagnostics-15-03152-s001.zip › Supplementary_Table_S3_Hyperparameters.pdf]

Supplementary Table S3. Hyperparameter Search Space and Selected Values for Machine-Learning Models

Model	Hyperparameter	Search Space / Grid	Selected Value
Random Forest (RF)	Number of trees (ntree)	{500}	500
Random Forest (RF)	Variables tried at each split (mtry)	{sqrt(p), p/3, p/2}	4
Stochastic Gradient Boosting (SGB)	Number of trees (n.trees)	{500, 1000}	1000
Stochastic Gradient Boosting (SGB)	Interaction depth	{2, 3}	2
Stochastic Gradient Boosting (SGB)	Shrinkage (learning rate)	{0.05, 0.1}	0.05
Stochastic Gradient Boosting (SGB)	Minimum observations in terminal node	{10, 20}	20
XGBoost	Number of boosting rounds (nrounds)	{300, 600}	600
XGBoost	Maximum tree depth (max_depth)	{3, 5}	3
XGBoost	Learning rate (eta)	{0.05, 0.1}	0.05
XGBoost	Subsample	{0.7, 0.9}	0.9
XGBoost	Column subsampling (colsample_bytree)	{0.7, 1.0}	0.7
